# Patients Can Administer Mobile Audio Recordings to Increase Knowledge in Advanced Prostate Cancer

**DOI:** 10.1002/cam4.70433

**Published:** 2024-11-21

**Authors:** Daniel H. Kwon, Lauren Trihy, Nika Darvish, Eliza Hearst, Saffanat Sumra, Hala T. Borno, Rohit Bose, Jonathan Chou, Ivan de Kouchkovsky, Arpita Desai, Brad Ekstrand, Terence Friedlander, Gurleen Kaur, Vadim S. Koshkin, Samantha Nesheiwat, Karen Sepucha, Eric J. Small, Rahul R. Aggarwal, Jeffrey Belkora

**Affiliations:** ^1^ Division of Hematology/Oncology, Department of Medicine University of California San Francisco California USA; ^2^ Helen Diller Family Comprehensive Cancer Center University of California San Francisco California USA; ^3^ Department of Surgery University of California San Francisco California USA; ^4^ Geisel School of Medicine Hanover New Hampshire USA; ^5^ Harvard Medical School Boston Massachusetts USA; ^6^ Institute for Health Policy Studies University of California San Francisco California USA

**Keywords:** implementation science, palliative care, patient education, patient knowledge, prostate cancer, recordings, shared decision‐making

## Abstract

**Introduction:**

Consultation audio recordings improve patient decision‐making but are underutilized. Patient‐administered recording apps on mobile devices may increase access, but implementation has not been evaluated.

**Methods:**

We conducted a single‐arm study delivering education, coaching, and reminders for patients to record their appointment using a mobile recording app. Patients had progressive, advanced prostate cancer and an upcoming appointment where the option of docetaxel would be discussed. We used the RE‐AIM framework for evaluation. Reach was the proportion of patients who participated. Effectiveness was change in informed decision‐making pre‐ vs. post‐appointment. We used a questionnaire evaluating patient knowledge about docetaxel (0%–100% correct) and the decisional conflict scale‐informed subscale (0 = feels extremely uninformed to 100 = extremely informed) to compare means using the paired *t*‐test. Adoption was the proportion of providers agreeing to be recorded. Implementation was coordinator adherence to intervention delivery. We conducted semistructured interviews with patients, caregivers, and providers to assess barriers, facilitators, and suggestions for recording implementation.

**Results:**

Of 102 patients approached, 50 (49%) patients participated. Mean age was 75 years, 38 (76%) were Non‐Hispanic White, and 43 (86%) had telehealth appointments. Knowledge increased from 44.7% to 49.5% (*p* = 0.019), particularly about palliative care (42% answering correctly to 60%, *p* = 0.035). Decisional conflict‐informed subscale increased from 48.9 to 70.9 (*p* < 0.001). Forty‐three patients (85%) made a recording, of whom 33 (77%) reported the recording helped treatment decision‐making. All 17 providers agreed to be recorded. Coordinator adherence was high. Multi‐level barriers, suggestions, and facilitators mostly related to intervention complexity and stakeholder compatibility.

**Conclusion:**

Patient‐administered audio recordings had a positive effect on decision‐making, particularly for palliative care awareness. For broader implementation, efforts should focus on revising institutional policies; teaching patients or caregivers to use existing recording functions on their devices; leveraging artificial intelligence for transcription and summarization; and integrating recording into telehealth technology and electronic patient portals.

**Trial Registration:** https://clinicaltrials.gov/study/NCT05127850

## 1 Introduction

Shared decision‐making, a collaborative process in which patients and providers partner to make well‐informed, preference‐concordant clinical care decisions, is a cornerstone of high‐quality, patient‐centered cancer care [1]. Shared decision‐making promotes patient autonomy, psychosocial outcomes, and uptake of evidence‐based treatments [2, 3].

Despite its value, in practice, shared decision‐making does not always occur. This is partly because patients recall a fraction of information discussed in oncology consultations due to the amount and complexity of information [4, 5, 6]. Consultation audio recordings are an evidence‐based intervention known to improve patient recall, thereby improving knowledge, decision‐making quality, and psychological outcomes [7, 8, 9, 10, 11, 12, 13, 14]. Despite the evidence of benefit, recordings are underutilized [15].

With increasing mobile device ownership, there has been growing interest in patients creating recordings using personal devices [16, 17, 18, 19]. Little is known about implementing recordings in the mobile health context, where the technological burden is shifted to patients. We previously conducted a pilot study assisting patients to use a mobile application to record consultations [20]. We found that patient‐administered recordings were a feasible, acceptable, and valued intervention that improved patient decision‐making. We also identified modifiable barriers to recording implementation. However, the study design was limited by a small sample size and narrow evaluation.

In this study, we refined the intervention to deliver patient‐administered recordings and conducted a single‐arm prospective study in patients with progressive, metastatic castration‐resistant prostate cancer (mCRPC). We evaluated the intervention using the RE‐AIM framework with the hypothesis that the intervention is associated with improved patient knowledge and decision‐making. To reduce the confounding that could be introduced by including multiple disease types and settings, we selected progressive mCRPC as the disease setting to study. Patients with progressive mCRPC have reasonably standardized treatment options and report a poor understanding of treatments [21, 22, 23, 24] that is exacerbated by cognitive impairment from androgen deprivation therapy [25] and may be alleviated by recordings.

## 2 Materials and Methods

### 2.1 Overall Study Design

We conducted a hybrid implementation‐effectiveness trial in which we tested the effects of recordings while evaluating implementation outcomes (NCT05127850) [26]. We used convergent‐parallel, mixed methods by collecting quantitative and qualitative data from patients, caregivers, and providers concurrently, then merging the data in analysis and interpretation [27].

### 2.2 Intervention

Based on feedback from our pilot study, we refined implementation strategies to facilitate patients creating their own consultation audio recordings using a mobile device (Figure S1) [20]. One week before their appointment, a clinical trial coordinator sent instructions to create, listen, and share an audio recording using a mobile device to consenting patients and/or designated caregivers. One day pre‐appointment, the coordinator coached patients/caregivers by telephone to ensure understanding of application use. The coordinator also automated two text message reminders: one pre‐appointment as a reminder to record and another post‐appointment as a reminder to listen. The study was approved by the UCSF Institutional Review Board.

### 2.3 Participants

We consecutively approached patients with progressive mCRPC being seen at the UCSF Genitourinary Medical Oncology clinic who met the following criteria: (1) English‐speaking, (2) access to a mobile device, (3) chemotherapy‐naïve, (4) upcoming appointment with an oncology provider who anticipated discussing docetaxel as a treatment option, and (5) the oncology provider consents to being recorded. UCSF's implicit policy on audio recordings is to defer to state law requiring all parties to consent to recordings. UCSF providers are free to accept or decline patient bids to record visits.

For consenting patients who designated a caregiver to assist with the recording, we invited the caregiver to participate in the post‐appointment interview.

Lastly, we invited consenting patients' providers who were being recorded to participate in a post‐appointment survey and interview.

### 2.4 Evaluation

We used RE‐AIM (Reach, Effectiveness, Adoption, Implementation, Maintenance), an implementation framework used to evaluate programs in a way that facilitates the translation of research to practice with quality, speed, equity, and population‐level impact in mind (Table S1) [28]. We did not evaluate maintenance given the short follow‐up period of the study.

### 2.5 Reach

Reach was the proportion and representativeness of approached patients who consented to participate. We gathered reasons for non‐participation. We collected demographic data from pre‐appointment surveys and the electronic health record (EHR) for descriptive summarization. We also identified barriers, facilitators, and suggestions for Reach in provider interviews, described below in Section 2.6.

### 2.6 Effectiveness

Effectiveness was the impact of the intervention on patient informed decision‐making. We measured change in two patient‐reported primary outcomes measured 1‐week pre‐ and 1‐week post‐intervention: (1) objective docetaxel knowledge and (2) informed subscale of Decisional‐Conflict Scale (DCS), a subjective measure of knowledge. We hypothesized that the means of both outcomes would increase pre‐ versus post‐intervention. Docetaxel knowledge was evaluated using a 19‐item investigator‐created questionnaire scored 0%–100% (Supporting Information 1). We developed the knowledge questionnaire adapting methods described by Sepucha et al. [29, 30, 31]; (Table S2) to evaluate key facts a patient with progressive mCRPC should know when making a decision that includes docetaxel. The DCS‐informed subscale contains three items on a 5‐point Likert scale, scored 0 (feels extremely uninformed) to 100 (feels extremely informed) [32]. A secondary endpoint was change in patient‐reported anxiety (PROMIS anxiety short form 4a) [33]. We used the paired two‐sample *t*‐test to compare pre/post means of the aforementioned outcomes. For each knowledge item, we also compared pre/post proportions of patients who responded correctly using McNemar's test; we did not correct for multiple tests as our analyses were exploratory. We used Stata v18 for analyses. *p* < 0.05 was deemed significant.

The post‐appointment survey contained process items of whether patients created, listened to, and shared the recording; helpfulness of intervention components (5‐point Likert scale); and ease and comfort of recording (5‐point Likert scale). It also contained patient‐reported recording usefulness (5‐point Likert scale), recording helpfulness in treatment decision‐making (5‐point Likert scale), satisfaction with the application (5‐point Likert scale), and whether they plan to make future recordings. We used the EHR to assess the proportion of patients who received docetaxel as their next line of systemic therapy.

One‐week post‐appointment, we conducted semistructured interviews with patients ± caregivers using an interview guide to understand their experience with the recording process, especially with respect to treatment decision‐making, and to elicit barriers, facilitators, and suggestions for patient‐administered recording implementation (Supporting Information 2).

For patient participants' providers, we administered a survey 1 day post‐appointment assessing three items on a 5‐point Likert scale: (1) recording interference with the appointment, (2) recording effect on patient‐provider relationship, and (3) recording effect on patient care. We also invited all UCSF‐affiliated genitourinary oncology providers to a semistructured interview investigating same topics as those in the patient guide (Supporting Information 3).

We recorded and transcribed all interviews. Two coders coded transcripts independently using the Critical Incident Technique [34]. We consecutively interviewed patients until data saturation was met, defined as no new major barriers/facilitators in two consecutive interviews. We labeled each critical incident as a barrier, facilitator, or suggestion then categorized it under one of Rogers' factors for innovation diffusion (relative advantage, compatibility, complexity, trialability, and observability) [35]. Full methodological details are in the COnsolidated criteria for REporting Qualitative research checklist (Table S3).

### 2.7 Adoption

Adoption was the proportion of oncology providers who agreed to be recorded. We also identified barriers, facilitators, and suggestions for provider and organizational adoption in the provider interviews.

### 2.8 Implementation

Implementation was the coordinator's adherence to per‐protocol intervention delivery and adaptations to the intervention.

## 3 Results

### 3.1 Reach

Of 102 patients approached from March 2022 to March 2024, 50 (49%) were enrolled. Mean age was 75 years, 38 (76%) were White, and 43 (86%) had a telehealth video appointment (Table 1). The most common reasons for non‐participation were too busy (*n* = 11), unreachable (*n* = 11), and too ill (*n* = 9; Figure S3 and Table 2). Age and race/ethnicity of participants were similar to those of non‐participants (Table S4).

**TABLE 1:** Baseline characteristics of 50 patient participants.

Characteristic	Mean (SD) or *n* (%)
Age at recorded appointment (years)	75 (7)
Race/ethnicity	
White/Non‐Hispanic	38 (76%)
Asian/Non‐Hispanic	5 (10%)
Black or African American/Non‐Hispanic	4 (8%)
Native Hawaiian or Pacific Islander/Non‐Hispanic	1 (2%)
White/Hispanic	1 (2%)
White/Unknown if Hispanic	1 (2%)
Highest education level	
Some high school	1 (2%)
High school graduate or equivalent	5 (10%)
Some college, no degree	14 (28%)
Associate degree	4 (8%)
Bachelor's degree	18 (36%)
Master's degree	4 (8%)
Professional or doctorate degree	4 (8%)
Annual household income	
< $50,000	7 (14%)
$50,000–$99,999	13 (26%)
$100,000–$149,999	13 (26%)
$150,000–$199,999	6 (12%)
≥ $200,000	5 (10%)
Choose not to say	6 (12%)
Driving distance from residence to UCSF (miles)	
< 10	9 (18%)
10–49	19 (38%)
50–99	11 (22%)
≥ 100	11 (22%)
Health literacy^a^	
Adequate	48 (96%)
Limited	2 (4%)
Baseline cognitive function T score^b^	
< 50 (below average)	20 (40%)
≥ 50 (at or above average)	30 (60%)
ECOG performance status	
0	29 (58%)
1	21 (42%)
Number of prior lines of systemic therapy in the castration‐resistant setting	
0	13 (26%)
1	17 (34%)
2	8 (16%)
3	7 (14%)
≥ 4	5 (10%)
Charlson comorbidity index	
8	9 (18%)
9	19 (38%)
10	13 (26%)
11	7 (14%)
12	1 (2%)
13	1 (2%)
Distant sites of prostate cancer	
Bone only	21 (42%)
Bone and lymph node	18 (36%)
Bone and visceral	3 (6%)
Lymph node only	3 (6%)
Lymph node and visceral	2 (4%)
Bone and lymph node and visceral	2 (4%)
Visceral only	1 (2%)
Type of progression	
PSA and radiographic	35 (70%)
PSA only	10 (20%)
Radiographic only	5 (10%)
Appointment type	
Video conference	43 (86%)
In‐person	6 (12%)
Telephone	1 (2%)
Recording application type	
Voice Memos (Apple/iOS)^c^	30 (60%)
Medcorder	10 (20%)
Voice Recorder (Samsung/Android)^c^	8 (16%)
Hi‐Q	1 (2%)
Unknown	1 (2%)
Ever made a recording of a doctor's visit	
Yes	6 (12%)
No	43 (86%)
Missing	1 (2%)
Previously found a health/wellness app useful in discussions with a healthcare provider	
Yes	24 (48%)
No	26 (52%)

^a^ Per Cancer Health Literacy Test (CHLT‐6).

^b^ Per PROMIS Cognitive Abilities Short Form 4a.

^c^ These applications came pre‐installed on the mobile device.

**TABLE 2:** Evaluation of patient‐administered mobile app recordings by patients, caregivers, and providers.

RE‐AIM domain	Key results	Rogers' domain^a^	Barriers	Facilitators	Suggestions
Reach (patient level)	49% patients participated in recording program	Relative advantage	Feels recording is unnecessary		
		Compatibility	Too busy to record Too ill to record Insufficient devices Not tech savvy App and data security concerns Does not like remote tools Does not want to be distracted during appointment Cancer topic and appointments already cause anxiety Scared to get provider consent to record Worried about legality of recordings Not aware recording an appointment is possible	Smartphone ownership Access to family smartphone Provider already consented to being recorded	Conduct outreach to patients Provide patients with recording devices Offer patients choice between clinic‐ versus patient‐administered recordings Have provider pre‐sanction consent Integrate recordings with already‐used technology, like telehealth platforms
		Complexity	App is too complicated	Presence of a caregiver	Automate and standardize recordings
		Observability			
Effectiveness (patient level)	Knowledge score about docetaxel improved from 45% to 50% (*p* = 0.019) Informed subscale of decisional conflict scale improved from 49 to 71 (*p* < 0.001) Of those reached: 86% created a recording 66% listened to a recording 77% found recording at least a little helpful in treatment decision‐making	Relative advantage	Already takes notes during appointment Family takes notes during appointment or attends appointments Recordings have more redundancies and distractions than notes Difficult to understand recording if audio quality is poor	App is free	
		Compatibility	Doesn't know benefit of recordings Nothing important discussed during appointment Not time to make a treatment decision Already knowledgeable and remembers everything provider says Forgot to record/listen due to poor memory or too busy Poor hearing Provider speaks too fast or quietly Poor provider communication Difficult to skip to most important part of recording Listening to recording brings up negative emotions Worried about provider privacy when sharing recordings Not tech savvy Old age Lack of tech support Telehealth is onerous and susceptible to tech issues Unstable phone placement	Understands benefits of recordings Important upcoming appointment Forgets what is discussed in appointments Provider openness to being recorded App prompts to ask provider for consent Prior recording experience App similarity to other apps Tech savvy Text reminders to record and listen Comfort with telehealth visits Easier to record telehealth visits ^b^ Cross‐references recordings with notes Recordings can be transcribed	Central location to place phone Auto‐label recordings with date and speaker names Add transcription and search functions to recording App summarizes recording Video recording Telehealth platform also auto‐records Automatically share recording with other providers
		Complexity	Difficult to find app Too many app options Redundant steps to start recording Unclear which button starts recording Difficult to access saved recording Easy to accidentally delete recording File too large to share Clutter from ads in app	Easy to find app in app store App is pre‐installed on phone Easy to install app App simplicity with few steps Caregiver makes recording Caregiver technical assistance Coordinator technical assistance Written and video instructions	Staff reminders to record Clinic automatically makes recording Recording automatically uploads to patient portal
		Observability		Reading app reviews	
Adoption (provider and system levels)	100% of providers agreed to be recorded	Relative advantage			
		Compatibility	Lack of awareness of evidence surrounding recordings Legal risks from misuse, misinterpretation, tampering sharing of recordings by patients to be used against providers Concern of making false promises during recorded appointments Provider discomfort or anxiety with being recorded Less candor when being recorded Recording leads to more patient questions Lack of knowledge of policies surrounding recordings Lack of staff/resources to deliver recordings for all	Awareness of evidence surrounding recordings Experience seeing patients struggle with information processing and decisions Belief that recordings have a wide range of benefits to patients and caregivers that outweigh risks Belief that recordings are particularly helpful for auditory learners, caregivers, elderly, and non‐English speakers Belief that recordings increase patient‐provider trust Belief that recordings augment informed consent Belief that being recorded should not affect information from providers; always speak as if being recorded Belief that more patient questions is beneficial Perception that recordings are commonly done Positive institutional culture surrounding recordings Lack of concern about legal ramifications Patients and providers providing consent to recordings	Educate providers on evidence for recordings Adapt recording delivery to serve wide range of socio‐economic, language, and technology backgrounds Record only important appointments Deliver recordings to patients who'd benefit most Create and enforce institution policies surrounding recordings Educate providers on institutional recording policies Ensure recordings cannot be replicated Reassure providers about protections for recordings Engage institution leadership and division stakeholders Create UCSF‐sanctioned app Add disclaimer about purposes of recording Centralize and secure storage of recordings at UCSF
		Complexity	Extra burden on patients, especially those who are older, tech illiterate, or socioeconomically disadvantaged Slowing down provider workflow		Simplify app Provide patients with hands‐on guidance Patient teach sessions Upload recordings to patient portal Automate text message reminders for patients to record and listen Streamline recording delivery so it does not impact provider workflows
		Observability	Lack of feedback from patients who recorded		Gather and report data on implementation and impact of audio recordings
Implementation (patient and staff level)		Relative advantage			
		Compatibility			
		Complexity	Maintaining up‐to‐date, complete instructions Scheduling coaching because hard to reach patient or appointment is rescheduled. Forgot to automate text message reminders for patients. Helping patients share recordings		
		Observability			

^a^ Barriers, facilitators, and suggestions were categorized according to Rogers' Diffusion of Innovation theory, which describes five key factors that influence the adoption of an innovation: Relative advantage (the degree to which an innovation is perceived as better than the idea or practice it replaces), Compatibility (consistency of the innovation with existing values, past experiences, and needs of potential adopters), Complexity (the degree to which an innovation is perceived as difficult to understand and use), Trialability (the degree to which an innovation can be experimented with on a limited basis before full adoption), and Observability (the degree to which the results of an innovation are visible to others). In this study, participants did not report factors relevant to Trialability.

^b^ Reasons telehealth visits were easier to recording were because it is more comfortable to record from home and phone placement is more physically secure.

Nine providers (56% of 16 approached) completed interviews. Though providers recognized that patient‐administered recording apps increase access to recordings compared to clinic‐administered methods, particularly with telehealth appointments, some worried about the technological burden for non‐tech‐savvy patients and that patients may be anxious to ask providers for consent to being recorded (Table 2). Suggestions included offering the intervention to all patients, providing patients a choice of patient‐administered or clinic‐administered recordings, providing patients with devices to record, standardizing recordings by embedding recordings in already‐used technology (like telehealth platforms), and having providers pre‐sanction recordings.

The more you can embed within the technology that they're already using to join the visit. You want to automate and streamline. Have a link embedded within the patient portal that allows them to just click one button and say, “I'm recording this.”—Provider 4.

### 3.2 Effectiveness

**FIGURE 1:**
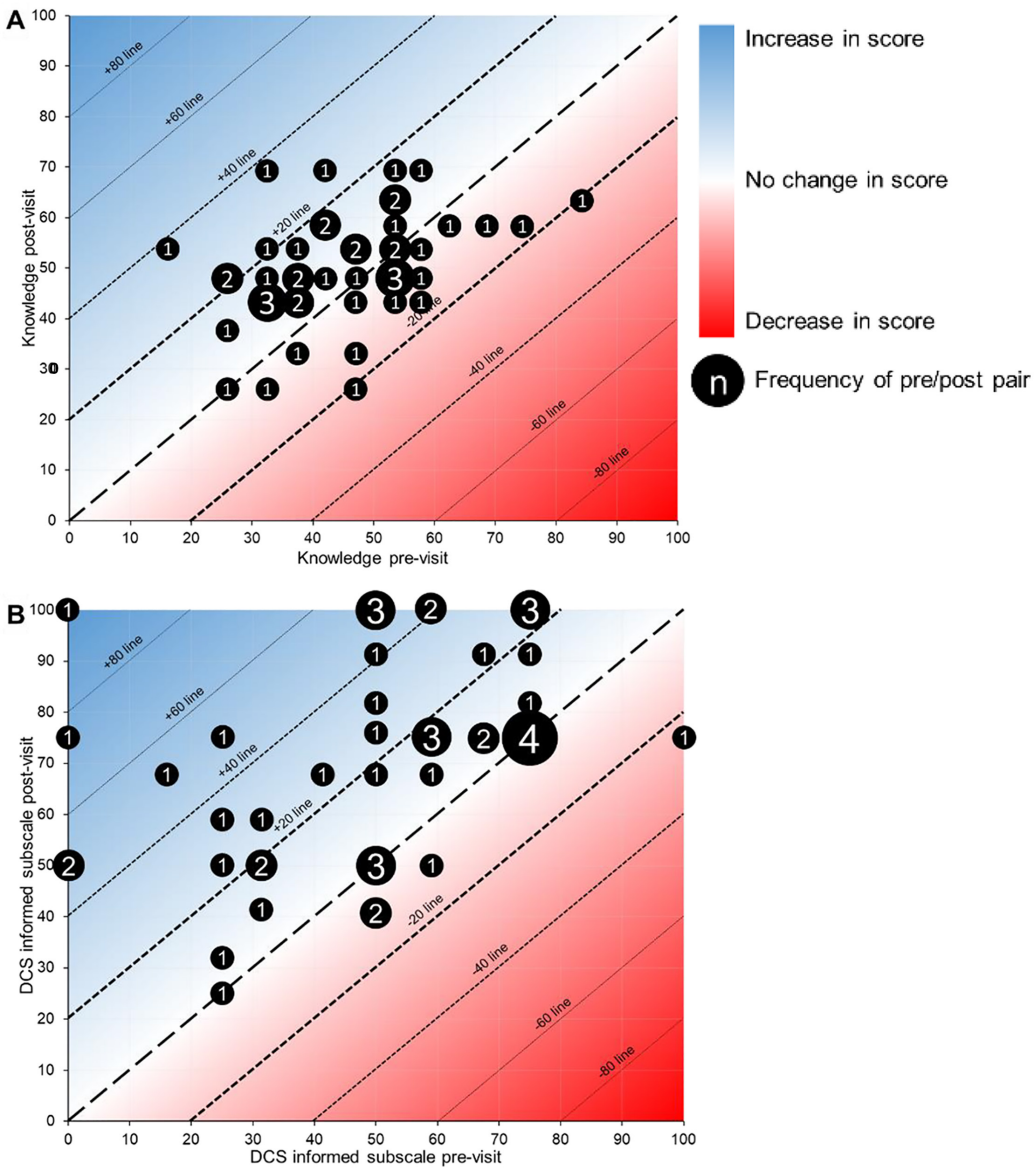
Weighted scatter plot showing pre/post changes in informed decision‐making and total knowledge scores. Diagonals denote change in score. Numerals above the mid‐diagonal represent a positive change in score, and those below represent a negative change. (A) Overall, the mean knowledge score increased from 44.7% to 49.5% (*p* = 0.019). Two patients did not complete the post‐appointment survey and were excluded from analysis. In sensitivity analyses in which we indicated that the two patients responded either as the least effective or most effective response for other patients with their baseline knowledge score, the results were not significantly different. (B) Overall, the mean DCS informed scale increased from 48.9 to 70.9 (*p* < 0.001). Three patients did not complete the post‐appointment survey and were excluded from analysis. In sensitivity analyses in which we indicated that the three patients responded either as the least effective or most effective response for other patients with the same baseline DCS score, the results were not significantly different.

**FIGURE 2:**
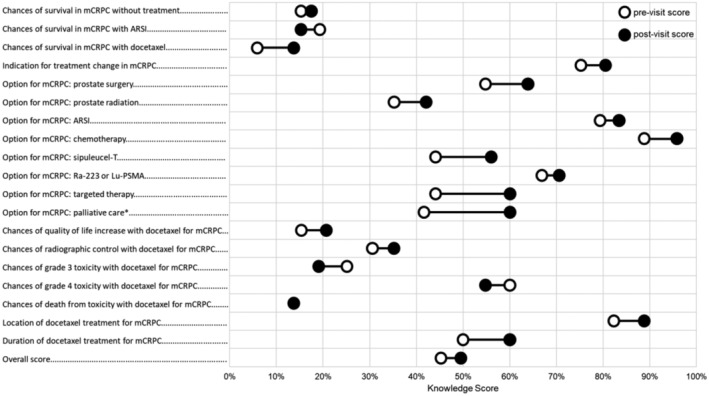
Pre/post changes in individual knowledge items. *Statistically significant difference (*p* < 0.050). There were 19 individual items that comprised the knowledge questionnaire based on key facts a person should know when consider docetaxel as a treatment option in mCRPC. ARSI = androgen receptor signaling inhibitor. mCRPC = metastatic castration‐resistant prostate cancer.

Patient knowledge increased from a mean pre‐appointment score of 44.7% to 49.5% post‐appointment, equivalent to one additional correctly answered item (*p* = 0.019; Figure 1). Of the 19 items in the knowledge questionnaire, one item had a significant increase in proportion of patients who responded correctly: palliative care is an option in mCRPC (42% to 60%, *p* = 0.035; Figure 2). The DCS informed subscale score increased from 48.9 to 70.9, equivalent to improving from feeling neither informed/uninformed to feeling somewhat informed (*p* < 0.001; Figure 1). There was no change in patient‐reported anxiety (*T*‐score 56.0 to 54.2, *p* = 0.140).

In subgroup analyses based on whether the patient listened to the recording, there was a greater improvement in knowledge among patients who listened (7% increase) compared to those who did not (1% decrease, *p* = 0.032). There were no differences in DCS informed subscale or anxiety between patients who listened and those who did not.

Twenty patients (40%) received docetaxel as their next line of systemic therapy. There were no differences in docetaxel receipt based on listening, and knowledge scores did not vary based on docetaxel receipt.

Table 3 describes the remaining patient‐reported recording outcomes. The most helpful recording support component was text message reminders. Forty‐one (85%) patients found it easy to make a recording. Forty‐three patients (85%) made a recording, and 33 (66%) listened to the recording. Among 43 patients who recorded, 37 (86%) patients found the recording at least a little useful and 33 (77%) at least a little helpful in treatment decision‐making.

**TABLE 3:** Recording‐related outcomes.

Process measures	*n* (%)
Recording support	
Written instructions were at least a little helpful	31 (65%)^a^
Video instructions were at least a little helpful	13 (27%)^a^
Text message reminders were at least a little helpful	37 (77%)^a^
Received help finding, installing, or using the recording app	13 (27%)^a^
Easy to install app (agree/strongly agree)	39 (81%)^a^
Easy to make recording (agree/strongly agree)	41 (85%)^a^
Recording process	
Made a recording	43 (86%)^b^
Listened to a recording	33 (66%)^c^
Shared recording	11 (22%)^d^
Comfortable making a recording (agree/strongly agree)	44 (92%)^a^
Outcome measures	*n* (%)
Recording usefulness	
Very useful	21 (42%)
Somewhat useful	10 (20%)
A little useful	6 (12%)
Not useful	5 (10%)
N/A didn't record	7 (14%)
Missing	1 (2%)
Recording helpfulness in making treatment decision	
Very helpful	12 (24%)
Somewhat helpful	13 (26%)
A little helpful	8 (16%)
Not helpful	9 (18%)
N/A didn't record	7 (14%)
Missing	1 (2%)
Satisfaction with using an app to record oncology visit	
Very satisfied	13 (26%)
Somewhat satisfied	22 (44%)
Neutral	12 (24%)
Somewhat dissatisfied	0
Very dissatisfied	1 (2%)^e^
Missing	2 (4%)
Plan to make recordings of appointments in the future	
Yes	43 (86%)
No	5 (10%)
Missing	2 (4%)

^a^ Denominator is 48 participants, as two participants did not complete the post‐survey.

^b^ Reasons for not making a recording include forgot to record (2), appointment was rescheduled (2), didn't install recording app (1), difficulty using app (1), and mobile device for recording was unavailable (1).

^c^ Reasons for not listening to recording include no need to listen (6), no time to listen (2), and forgot to listen (2).

^d^ Recipients included the participant's partner (10), child (2), friend (2), and other family (2).

^e^ Patient was upset about provider communication during the visit, not the recording or app.

In post‐appointment surveys of 17 providers across 48 evaluable encounters, recordings interfered little with the appointment and had a mostly positive effect on the provider–patient relationship and patient care (Table 4).

**TABLE 4:** Provider‐reported outcomes across encounters of 48 evaluable patients.

Outcome measure	*n* (%)
How much did the audio recording interfere with the appointment?	
No interference	46 (96%)
A little interference	1 (2%)
Some interference	1 (2%)
Significant interference	0
How did the audio recording affect the provider–patient relationship?	
Positively	42 (88%)
No change	6 (13%)
Negatively	0
The audio recording overall enhanced the patient's care.	
Strongly agree	2 (4%)
Agree	17 (35%)
Neither agree nor disagree	28 (58%)
Disagree	1 (2%)^a^
Strongly disagree	0

^a^ One provider reported that being recorded changes the nature of the interaction. There is a “loss of frank exchange that might be possible in the absence of recording.”

For qualitative data, we interviewed 30 patients and 10 caregivers and integrated the results with those from provider interviews. Interviewees described successes with the recording process. These ranged from patients and providers experiencing a seamless, non‐intrusive recording process, including in telehealth appointments, to numerous ways in which recordings were helpful (Table S3). Key patient benefits were feeling more engaged during the appointment; picking up information that was missed, misheard, forgotten, or mis‐remembered; processing information and emotions; increasing confidence in decisions; feeling reassured that there is an accurate, unbiased record; helping communicate with family members; and acting on provider recommendations that they had forgotten.

The recording can be a handy resource in case there is a dispute about what you discussed… Listening to it multiple times can help you process the diagnosis and what the options are. Because you can go through it again and process it much slower. It gives you more time to decide, and then you are more certain that you made a better, more thoughtful decision. I wish I had a recording when I was first diagnosed with cancer, because the minute you receive that diagnosis, you're shell shocked. Everything goes in one ear and out the other. I really don't even know what the doctor said. The only thing you hear is cancer.—Caregiver of Patient 15.

Providers reported that recordings made them more thoughtful with their choice of words and reassured them that that patients would remember important information they communicated.

However, there were also problems, including the recording process being cumbersome, accidentally deleting the recording, and observing poor audio quality. One new provider reported feeling anxious because of fear of misspeaking while being recorded.

The Effectiveness row in Table 2 describes barriers, facilitators, and suggestions for effective recording delivery. Some patients were too forgetful, tech illiterate, busy, or anxious to make or listen to the recording. Text reminders and caregiver/coordinator assistance helped patients overcome these barriers.

“I don't think I would've been able to do it without my son's help. He actually got the recording then sent it to me and I listened to it.”—Patient 31.

Several patients did not experience any benefit from recordings because they took thorough notes, remembered what their provider communicated, or did not make a decision during the appointment. Recognizing that the appointment was important encouraged listening:

We knew that it was a really important appointment. So we wanted to listen to it again, even though we knew it was going to be difficult to listen to.—Patient 19.

As most barriers and facilitators were in the Compatibility and Complexity categories, suggestions focused on adding specific features to apps to address individual concerns (e.g., data privacy) and preferences (e.g., adding search and transcription functions), and simplifying recordings through automation.

[I'd like] a better app. A secure transcription service would be nice. The ability to add your own timestamps so that I would come right back to that feedback. Sharing, too. One question is whether it's a privacy concern for the physician. It would be nice to have it in [patient portal] so it's all in one place.—Patient 22.

### 3.3 Adoption

All 17 providers agreed to be recorded; no provider declined being recorded. In qualitative interviews with providers, we identified barriers, facilitators, and suggestions for broader adoption of patient‐administered recordings by providers and healthcare systems (Table 2). Key barriers included unawareness of the benefits of recordings and institutional policies surrounding recordings, as well as concerns about recording tampering and misinterpretation, increase in workload, burdening patients, and inadequate resources to help all patients with recordings. Separate from their experiences in this study, providers reported recordings being misused or misinterpreted:

One of the negative downstreams of recordings I have experienced is another patient having heard the same recording: "Hey, my best friend Bob had this recording and you're doing this for him, how come you're not doing it for me?" The answer is obviously pretty complicated. We are dependent on individual circumstances.—Provider 9.

A key facilitator was the belief that the benefits of recordings outweigh potential risks. Suggestions for broad adoption were educating providers on the evidence and policies surrounding recordings, engaging stakeholders, concentrating recording delivery to targeted patients and appointments, developing an institution‐sanctioned recording app, and gathering data during implementation.

It would put less onus on us if you see an official policy, which I don't even know if they do. That gives the clinician a bit of cover because it has always felt a little uncomfortable when the patient asks [whether they can record].—Provider 5.

### 3.4 Implementation

There were no irregularities with the coordinator's per‐protocol adherence to intervention delivery. The primary difficulty was difficulty helping patients share their recording because the recording file sizes were large.

In terms of adaptations to the intervention during implementation, we had initially recommended that patients use Medcorder, an app specifically created to record medical appointments. However, 4 months into study activation, Medcorder was discontinued. Instead, we revised our instructions to help patients find an app of their choice. Most patients chose a pre‐installed app (Table 1).

## 4 Discussion

In this study, patient‐administered audio recording implementation was associated with improved decision‐making in patients with progressive mCRPC. Patients, caregivers, and providers found recordings to be helpful in many ways, including treatment decision‐making. We identified barriers, facilitators, and suggestions for widespread, equitable, and effective implementation of recordings to help patients with cancer make informed decisions.

Our study improved its primary outcomes of improving patient knowledge and informed decision‐making. This is consistent with two smaller studies of patient‐administered recording apps and randomized studies of clinic‐administered recordings [13, 16, 20]. In particular, patient awareness of palliative care increased, which has not been previously reported. Although it is unclear why this occurred, it is possible that being recorded may have prompted providers to be more thoughtful and mention palliative care, and listening to recordings may have reminded patients of palliative care. This finding is important as early palliative care may lead to better quality of life, less aggressive end‐of‐life care, and longer survival; the American Society of Clinical Oncology recommends integrating palliative care into routine oncology care [36]. It is similarly unclear why more patients chose docetaxel (40%) compared to historical figures (13%–27%) [37, 38, 39]. This may be due to difference in patient characteristics, provider practice patterns, or more accurate patient perceptions of chemotherapy's benefits and harms due to recordings.

In this single‐arm study, we cannot prove causality between patient‐administered audio recordings implementation and improved decision‐making, since similar changes may have occurred without the recording. However, our subgroup analysis suggests that patients who created and listened to recordings particularly benefited compared to those who did not. Furthermore, numerous randomized clinical trials have demonstrated benefits of clinic‐administered recordings that align with benefits reported in our patient interviews [14]. Most patients created and listened to recordings and found recordings helpful in both surveys and interviews. Nearly all providers reported little‐to‐no negative effects of recordings on their workflow or patient relationships. Altogether, our findings suggest that patient‐administered recordings produce similar benefits as those of clinic‐administered recordings with minimal drawbacks.

Patient‐administered recordings may have additional benefits of giving patients a greater sense of control and providing features such as transcription, reminders, and sharing with others. The original study intervention included a recording app tailored for medical appointments (Medcorder) containing these features. However, the app publisher discontinued support for this app, and we pivoted to training patients to use free apps. Patients responded well to simple, pre‐installed apps like Voice Memos on iOS, and most were able to successfully make recordings.

Many barriers and facilitators for the effectiveness of patient‐administered recording overlapped with those of clinic‐administered recordings: listening being a source of distress, being too busy or ill, perceived value of recordings, legal/privacy concerns, and required resources and technology. Unique to patient‐administered recordings, patient ability to find/install/use a recording app was the most cited barrier, particularly for telehealth visits. This was not surprising given the generally older prostate cancer population (mean age = 75 years) that is less familiar with technology. Caregiver and coordinator assistance was critical in helping non‐tech‐savvy patients, as were multi‐modal instructional materials and automated text message reminders. We also uncovered previously unreported barriers: lower relative advantage compared to note‐taking, recording containing insubstantial or poorly communicated information, and complexity in sharing the recording with others due to the large file size.

With respect to Reach, half of the patients we approached declined participating in the study, higher than in the pilot study we conducted (26%). This difference likely stems from the inclusion criterion of docetaxel being considered as a treatment, which selects for patients who may be too ill to participate. Some patients declined participating solely because of study procedures (e.g., too busy to complete surveys). Accounting for such study‐related reasons for non‐participation, we estimate the proportion of patients who would want to create a recording in a real‐world setting may be 55%–88%. Most barriers/facilitators for Reach related to the need to obtain consent from providers to record them, having the technical knowledge/skills and sufficient devices to record, and the severity of patients' illness or overall business, all of which are more pronounced when patients carry the burden of creating recordings.

In terms of representativeness, our patient sample was well‐educated, mostly White, and English‐speaking, limiting the generalizability of our findings. However, we would expect non‐White patients to be reached similarly as White patients, as cell phone ownership varies minimally based on race (96%–99%) [40] We also recruited only English‐speaking patients as study instruments were available only in English, but implementation should not discriminate based on language. A study by Lipson‐Smith, et al. suggested that non‐English speakers benefit from recordings, and a randomized pilot study in patients with limited English proficiency is ongoing [41, 42]. Many providers proposed that non‐English‐speaking patients and families would particularly benefit from recordings, for example, they could seek transcriptions or translations of the recording for enhanced understanding.

Providers generally promoted widespread adoption of patient‐administered recordings, believing benefits outweighed risks. Many barriers and facilitators were similar to what has been found in clinic‐administered recordings: perceived value, fear of distressing patients, impact on workflow and communication, lack of feedback from patients about recordings, patients tampering or misinterpreting recordings, and legal/privacy risks [13, 43, 44]. There were also concerns about scalability of delivering hands‐on training. We also identified previously unreported barriers: fears of recordings leading to more questions, institutional culture, and absence of institutional policies.

To increase Reach, Effectiveness, Adoption, and Implementation of patient‐administered recordings, health care systems should consider a number of strategies. Systems could conduct outreach for patients whose providers have “pre‐sanctioned” being recorded. To assist patients in making recordings, strategies include engaging caregivers, providing education (particularly awareness of built‐in voice recording apps) and technical assistance, lending devices, and offering clinic‐administered recordings. To minimize burdens associated with these measures, systems could automate education and reminders for only key appointments. Systems should also leverage technology by integrating recording technology within institution‐sanctioned patient portal apps or telehealth platforms, and using artificial intelligence (AI) for automatic transcription/summarization, with caution to inaccuracies and hallucinations. For example, audio recordings used for AI‐based scribing could be sent to patients [45]. Lastly, to promote adoption at the provider and organizational levels, we recommend educating stakeholders about the benefits of recording, creating policies governing recordings with stakeholder input, engaging clinical champions, and evaluating and reporting on implementation in real‐time—all essential components of a learning health system [46].

Limitations of our study include the pre/post study design, using an investigator‐created questionnaire without psychometric testing, and racial under‐representation of patient participants, as described above. Strengths of the study include the multiple types of participants and use of an implementation framework for evaluation.

## 5 Conclusion

Our intervention to implement patient‐administered consultation audio recording apps was associated with improvements in patient knowledge, particularly palliative care awareness, and decision‐making. Most patients, caregivers, and providers found patient‐administered recordings to be helpful and non‐intrusive, including in telehealth appointments. For wide‐reaching, equitable, effective, and highly adopted implementation of recordings, efforts should focus on automated outreach, patient training and assistance, stakeholder education about the evidence, stakeholder engagement, technology development and integration, continuous evaluation, and policy creation.

## Author Contributions

**Daniel H. Kwon:** conceptualization (lead), data curation (lead), formal analysis (lead), funding acquisition (lead), investigation (lead), methodology (lead), project administration (lead), resources (lead), software (lead), supervision (lead), visualization (lead), writing – original draft (lead), writing – review and editing (lead). **Lauren Trihy:** data curation (supporting), formal analysis (supporting), writing – review and editing (equal). **Nika Darvish:** data curation (supporting), formal analysis (supporting). **Eliza Hearst:** data curation (supporting), formal analysis (supporting). **Saffanat Sumra:** data curation (supporting), formal analysis (supporting). **Hala T. Borno:** conceptualization (supporting), writing – review and editing (supporting). **Rohit Bose:** writing – review and editing (equal). **Jonathan Chou:** writing – review and editing (equal). **Ivan de Kouchkovsky:** writing – review and editing (equal). **Arpita Desai:** writing – review and editing (equal). **Brad Ekstrand:** writing – review and editing (equal). **Terence Friedlander:** writing – review and editing (equal). **Gurleen Kaur:** formal analysis (supporting), writing – review and editing (supporting). **Vadim S. Koshkin:** writing – review and editing (equal). **Samantha Nesheiwat:** formal analysis (supporting). **Karen Sepucha:** methodology (supporting), writing – review and editing (equal). **Eric J. Small:** writing – review and editing (equal). **Rahul R. Aggarwal:** conceptualization (equal), funding acquisition (equal), resources (equal), supervision (lead), writing – review and editing (equal). **Jeffrey Belkora:** conceptualization (equal), methodology (equal), supervision (lead), writing – review and editing (equal).

## Ethics Statement

The study was reviewed and approved by the University of California, San Francisco Institutional Review Board.

## Consent

All participants provided informed consent prior to study procedures.

## Conflicts of Interest

Dr. Sepucha reports grants to her institution from PCORI, AHRQ, and NIA outside the current project and consulting contract through her institution with Blue Cross Blue Shield Massachusetts outside current project.

## Precis

Patient‐administered consultation audio recording apps were delivered with high adoption and adherence, leading to a positive effect on patient knowledge and decision‐making. Implementation efforts should focus on automated outreach, patient training & and assistance, stakeholder education about the evidence, stakeholder engagement, technology development & and integration, continuous evaluation, and policy creation.

## Supporting information


Data S1.

## Data Availability

The authors can make the full de‐identified dataset available to researchers upon reasonable request.
