# Supplementary material for: Patients Can Administer Mobile Audio Recordings to Increase Knowledge in Advanced Prostate Cancer
Source: Cancer Med. 2024 Nov 21;13(22):e70433. doi: 10.1002/cam4.70433 (PMC11579662; doi:10.1002/cam4.70433)
Supplement: Supplementary file 1 — Data S1. [file CAM4-13-e70433-s001.docx]

**Table of Contents**

[**Figure 1. Intervention Description** 2](#_Toc181948131)

[**Table 1. Measures for RE-AIM Evaluation** 3](#_Toc181948132)

[**Document 1. Docetaxel Knowledge Test** 4](#_Toc181948133)

[**Table 2. Adapted development process for docetaxel knowledge test development** 12](#_Toc181948134)

[**Document 2. Patient / caregiver semi-structured interview guide** 13](#_Toc181948135)

[**Document 3. Provider interview guide** 17](#_Toc181948136)

[**Table 3. COnsolidated criteria for REporting Qualitative research** 19](#_Toc181948137)

[**Figure 2. Participant Flow Diagram** 24](#_Toc181948138)

[**Table 4. Comparison of Evaluable versus Non-Responding or Unevaluable Participants’ Baseline Characteristics** 25](#_Toc181948139)

[**Table 3. Patient- ,Caregiver-, and Provider-reported Benefits to Self-Administered Recordings** 26](#_Toc181948140)

# **Figure 1. Intervention Description**


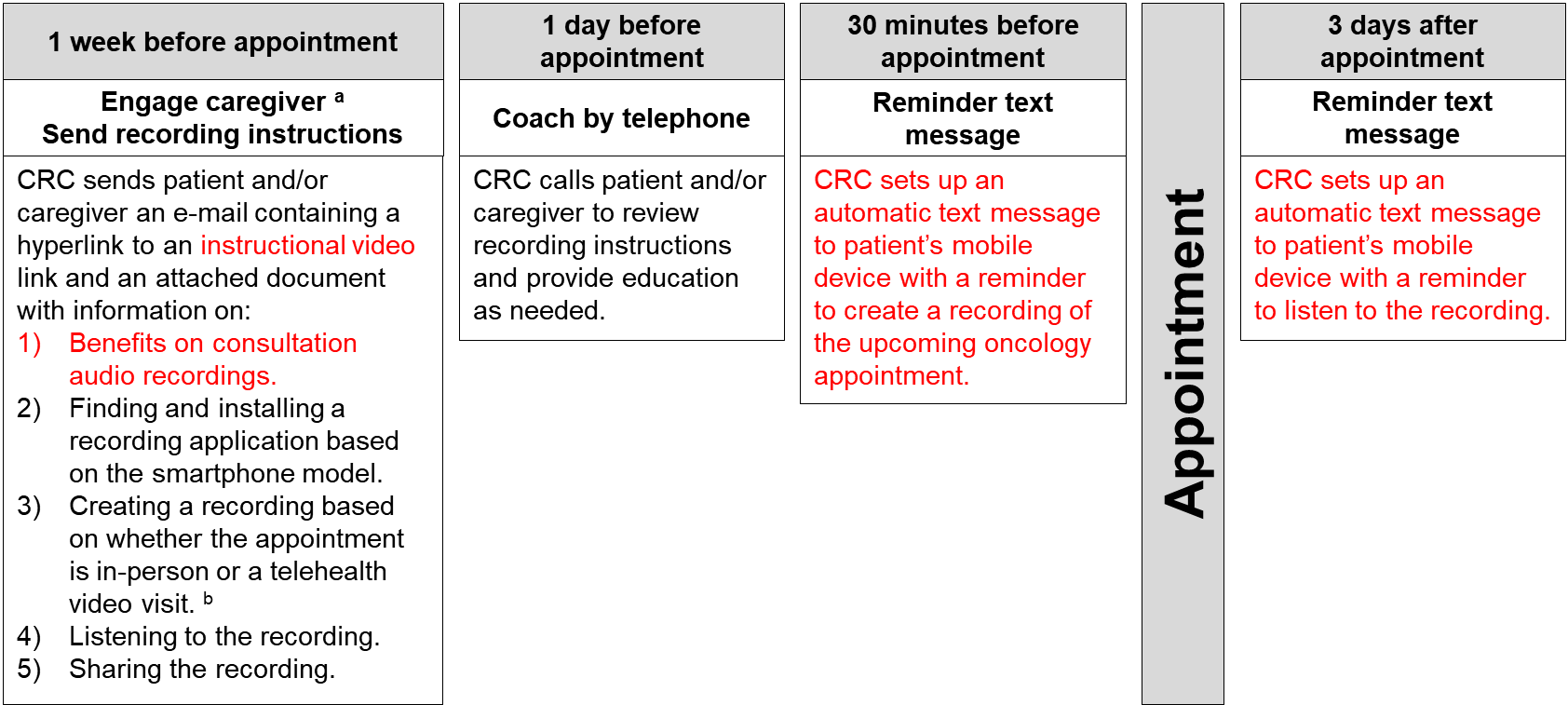


^a^ CRC asked the patient whether they would like to designate a caregiver to assist with the recording process.

^b^ For video visits, the document instructed patients to place their mobile device running the recording application next to the speakers of the device running the video visit.

Note: Red text denotes changes to interventions based on barriers and facilitators identified in a pilot study. CRC = Clinical Research Coordinator.

# **Table 1. Measures for RE-AIM Evaluation**

| **Domain** | **Quantitative** | | **Qualitative** | | **Study** **logs** |
| --- | --- | --- | --- | --- | --- |
|  | **Patient** **survey** | **Provider survey** | **Patient± caregiver interview** | **Provider interview** |  |
| **Reach** |  |  |  | x | x |
| **Effectiveness** | x | x | x | x |  |
| **Adoption** |  |  |  | x |  |
| **Implementation** |  |  |  |  | x |
| **Maintenance** | Not assessed | | | | |

# **Document 1. Docetaxel Knowledge Test**

First, we are interested in your knowledge of the following prostate cancer facts. These are facts that are important for a man with metastatic hormone-resistant prostate cancer who is making a decision that includes docetaxel (Taxotere) chemotherapy.

- Metastatic means the cancer has spread from the prostate to distant places like the bones, lungs, liver, or distant lymph nodes.
- Hormone-resistant means the cancer is resistant to hormone treatments that lower testosterone. The cancer has grown despite medicines like Lupron, Eligard, Firmagon, Orgovyx, and other similar medicines.

You will be asked about 12 facts that cover prognosis, options, risks, benefits, and logistics. Many of the facts are statistics based on research. As a result, the facts include time frames that are dictated by what data are available (for example, survival at 4 years).

Lastly, note that the questions are NOT about your particular prostate cancer, but for the "average man" in the given situation.

1. Picture a theater seating 100 men who have just been diagnosed with metastatic hormone-resistant prostate cancer. Assume that all the men decide to receive no cancer treatment.

Four years later, how many men will be alive?

Slide the slider to indicate a number between 0 and 100. (Remember, first click on the slider to activate it).

===========================

For coding purposes, the correct answer is 1 and incorrect 0.

1: <5

0: 5 or greater

*Technically correct answer: 0-1*

*Oudard S, Fizazi K, Sengeløv L, et al. Cabazitaxel versus docetaxel as first-line therapy for patients with metastatic castration-resistant prostate cancer: a randomized phase III trial—firstana. JCO. 2017;35(28):3189-3197.*

2. Picture a theater seating 100 men who have just been diagnosed with metastatic hormone-resistant prostate cancer. None of the men have ever taken a novel hormone treatment like abiraterone (Zytiga) or enzalutamide (Xtandi). Assume that all the men decide to start either abiraterone (Zytiga) or enzalutamide (Xtandi). 

Four years later, how many men will be alive?

Slide the slider to indicate a number between 0 and 100.

===========================

For coding purposes, the correct answer is 1 and incorrect 0.

1: 25 to 45

0: < 25 or > 45

*Technically correct answer: 35*

*Ryan CJ, Smith MR, Fizazi K, et al. Abiraterone acetate plus prednisone versus placebo plus prednisone in chemotherapy-naive men with metastatic castration-resistant prostate cancer (COU-AA-302): final overall survival analysis of a randomised, double-blind, placebo-controlled phase 3 study. Lancet Oncol. 2015;16(2):152-60.*

*Beer TM, Armstrong AJ, Rathkopf D, et al. Enzalutamide in Men with Chemotherapy-naïve Metastatic Castration-resistant Prostate Cancer: Extended Analysis of the Phase 3 PREVAIL Study. Eur Urol. 2017;71(2):151-154.*

3. Picture a theater seating 100 men who have just been diagnosed with metastatic hormone-resistant prostate cancer. None of the men have started chemotherapy yet. Assume that all the men decide to start docetaxel (Taxotere) every 3 weeks.

Four years later, how many men will be alive?

Slide the slider to indicate a number between 0 and 100.

===========================

For coding purposes, the correct answer is 1 and incorrect 0.

1: 8 to 28

0: < 8 or > 28

*Technically correct answer: 18 (data censored)*

*Oudard S, Fizazi K, Sengeløv L, et al. Cabazitaxel versus docetaxel as first-line therapy for patients with metastatic castration-resistant prostate cancer: a randomized phase III trial— FIRSTANA. JCO. 2017;35(28):3189-3197.*

**The following questions are about treatment options.**

4. Which of the following are standard, recommended treatment options for metastatic hormone-resistant prostate cancer?

Check all that apply

Note: this is not a complete list of options.

€ Prostate surgery

€ Chemotherapy (infusions like docetaxel, Taxotere, cabazitaxel, and Jevtana)

€ Prostate radiation

€ Novel hormone treatment (pills like abiraterone, Zytiga, enzalutamide, or Xtandi)

€ Palliative care (treating symptoms related to cancer)

€ Immunotherapy (cancer vaccine like Sipuleucel-T or Provenge)

€ Radioactive infusion (infusions like Radium-223, Xofigo or Lutetium-PSMA)

€ Targeted treatment based on genetic testing (pills like olaparib or Lynparza)

===========================

For coding purposes, each option gets either a correct answer of 1 and incorrect of 0.

1: If checked for chemotherapy, novel hormone treatment, palliative care, immunotherapy, radioactive infusion, and targeted treatment based on genetic testing
If unchecked for prostate surgery and prostate radiation.

0: All other responses

**The following questions are about docetaxel (Taxotere) chemotherapy.**

5. Picture a theater seating 100 men who have metastatic hormone-resistant prostate cancer. None of the men have received chemotherapy yet.

Assume all the men report their baseline quality of life score. Then, they all take docetaxel chemotherapy every 3 weeks.

Four months after starting docetaxel, how many men will have seen their quality of life score increase from baseline?

Slide the slider to indicate a number between 0 and 100.

===========================

For coding purposes, the correct answer is 1 and incorrect 0.

1: 17 to 37

0: < 17 or > 37

*Technically correct answer: 27*

*Caffo O, Sava T, Comploj E, et al. Impact of docetaxel-based chemotherapy on quality of life of patients with castration-resistant prostate cancer: results from a prospective phase II randomized trial. BJU Int. 2011;108(11):1825-32.*

6. Picture a theater seating 100 men who have metastatic hormone-resistant prostate cancer. None of the men have received chemotherapy yet.

Assume all the men get baseline CT and bone scans. Then, they all take docetaxel chemotherapy every 3 weeks. They get scans every 3 months.

Twelve months after starting docetaxel,  how many men will have seen their cancer controlled on CT and bone scans?

Note: "controlled" means that scans show the cancer has not grown.

Slide the slider to indicate a number between 0 and 100.

===========================

For coding purposes, the correct answer is 1 and incorrect 0.

1: 40 to 60

0: < 40 or > 60

*Technically correct answer: 50*

*Oudard S, Fizazi K, Sengeløv L, et al. Cabazitaxel versus docetaxel as first-line therapy for patients with metastatic castration-resistant prostate cancer: a randomized phase III trial— FIRSTANA. JCO. 2017;35(28):3189-3197.*

7. Out of the 100 men, how many will die from a docetaxel side effect?

Slide the slider to indicate a number between 0 and 100.

===========================

For coding purposes, the correct answer is 1 and incorrect 0.

1: < 5

0: ≥ 5

*Technically correct answer: 1-2*

*Oudard S, Fizazi K, Sengeløv L, et al. Cabazitaxel versus docetaxel as first-line therapy for patients with metastatic castration-resistant prostate cancer: a randomized phase III trial—FIRSTANA. JCO. 2017;35(28):3189-3197.*

8. Out of the 100 men, how many will be experience a Grade 3 Side Effect from docetaxel?

Note: "Grade 3 Side Effect" is a side effect that is any one of the following:

- Limits ability to care for yourself. For example, feeling so tired that one can't get dressed.
- Results in being seriously disabled. For example, nerve injury to the feet that requires one to permanently need a wheelchair.
- Requires hospitalization. For example, pneumonia that requires receiving intravenous antibiotics in the hospital.

Slide the slider to indicate a number between 0 and 100.

===========================

For coding purposes, the correct answer is 1 and incorrect 0.

1: 30 to 50

0: < 30 or > 50

*Technically correct answer: 40*

*Araujo JC, Trudel GC, Saad F, et al. Docetaxel and dasatinib or placebo in men with metastatic castration-resistant prostate cancer (Ready): a randomised, double-blind phase 3 trial. The Lancet Oncology. 2013;14(13):1307-1316.*

9. Out of the 100 men, how many will experience a Grade 4 Side Effect from docetaxel?

Note: a "Grade 4 Side Effect" is a side effect that is life-threatening and requires an urgent intervention. For example, lung injury that requires a breathing ventilator machine in an intensive care unit.

Slide the slider to indicate a number between 0 and 100.

===========================

For coding purposes, the correct answer is 1 and incorrect 0.

1: 5 to 25

0: < 5 or > 25

*Technically correct answer: 15*

*Araujo JC, Trudel GC, Saad F, et al. Docetaxel and dasatinib or placebo in men with metastatic castration-resistant prostate cancer (Ready): a randomised, double-blind phase 3 trial. The Lancet Oncology. 2013;14(13):1307-1316.*

**Please answer the remaining three questions**

10. For a man with metastatic hormone-resistant prostate cancer, how long do doctors usually recommend that should docetaxel be given?

Select one correct answer

O As long as the benefits of docetaxel outweigh the side effects.

O 4 to 7 months (3 infusions)

O 8 to 12 months (6 infusions)

O None of the above

===========================

Correct answer is “As long as the benefits of docetaxel outweigh the side effects.”

11. For a man with metastatic hormone-resistant prostate cancer, when should cancer treatment be changed to another treatment?

Select one correct answer

O When the PSA goes up two or more times

O When scans show that the cancer has disappeared

O When scans show that the cancer is growing

O After 6 months of treatment

===========================

Correct answer is “When scans show that the cancer is growing”

12. Where do patients usually receive docetaxel?

Select one correct answer

O In a hospital during a hospital stay

O In an outpatient infusion center

O At home

O Any of the above

===========================

Correct answer is “In an outpatient infusion center”

**Scoring Instructions**

1. Sum the number of points for all items, and then divide by 19.
2. Final score is a percentage from 0-100%.

# **Table 2. Adapted development process for docetaxel knowledge test development**

| **Phase of instrument development** | **Steps** | **Result** |
| --- | --- | --- |
| 1. Identification of key facts | - Review clinical evidence and decision-making literature for chemotherapy in metastatic castration-resistant prostate cancer (mCRPC). - Conduct semi-structured interviews with patients (N=6/7 = 86% response rate) with mCRPC who had received docetaxel and genitourinary medical oncologists (N=6/8 = 75% response rate) to identify all key facts about prostate cancer, docetaxel risks/benefits/logistics, alternatives, and misconceptions that a patient with mCRPC should know when considering docetaxel. - Identify candidate facts, cite evidence, and review with medical experts (DK and RA) and decision‐making experts (DK, JB, and KS). - Draft facts into survey item format. | - Initial set of 68 facts after removing duplicates and facts that were mentioned only once. - 29 facts after ranking facts on materiality, variability across likely patient answers, and availability of evidence to indicate a correct response. - 27 survey items after 5 facts were removed because of similarities with other facts and after 2 distractor facts were added. |
| 2. Drafting of questions | - Conduct cognitive testing of survey items with patients (N=4) to ensure understandability. - Conduct cross‐sectional surveys of patients with mCRPC who had received docetaxel in the past 12 months (N=22/47 = 47% response rate) and providers (N=11/14 = 79% response rate) to assess accuracy, importance, and completeness of candidate items. - Revise items based on literacy review by experts (DK, JB, and KS). - Revise items based on medical review by oncologists (DK and RA). - Select final items for testing and validation. | - Revised survey wording to optimize patient understanding and accuracy of items. - Final set of 19 survey items after removing least important facts and grouping docetaxel toxicity items into general adverse events. |
| 3. Testing and validation of instrument | - Field test with patients to examine psychometric properties (test-retest reliability, construct validity, internal consistency) and clinical sensibility (acceptability, feasibility). - Evaluate performance in diverse samples of patients. - Review items for accuracy and completeness on annual basis, including patient and provider input as needed . | - Not yet performed. |

# **Document 2. Patient / caregiver semi-structured interview guide**

**Preface**

I’d like to start with some background information. We know that men with prostate cancer are often given a lot of information during their appointments, and this can be overwhelming. As a result, many men don’t remember everything from their visits that they would like. This can make it difficult to make well-informed decisions.

We believe a recording app can be helpful for this problem, so we instructed you on using one. In this interview, we want to learn about your experience using this recording app. The information that you share will help us support all men with prostate cancer make well-informed decisions.

This is not a test. We are interested in your experience. There are no right or wrong answers, and it’s actually helpful for us if you disagree with me or have something negative to say.

I have now started recording.

**1. Decision-making**

Before we talk about the app, I’d last to talk about informed decision-making. By informed decision-making, I mean making a decision based on valid information and careful consideration of your personal goals and priorities.

Do you agree with the goal that men with prostate cancer should make informed decisions about treatments? Tell me more.

At your last appointment with [appointment provider] at UCSF, did you discuss making a treatment decision for the prostate cancer?

*If yes*:

- - Very briefly, can you tell me about the decision you discussed?
  - What stage you are at with the decision? *E.g. has decided or still deciding.*
    - *If already made the decision:* Do you feel like you made an informed decision? What makes you say that?
    - *If still making the decision:* Do you feel like you are on track to make an informed decision? What makes you say that?
  - What if anything is making this decision hard for you?
    - Has anything else made it hard?
  - What if anything has made the decision easier for you?
    - Was anything else helpful?
  - Can you think of anything that could help [mentioned difficulties]?

*If no*:

- - I see. Did you talk at all about future potential treatments?
    - If yes: What did you talk about?
    - If no: Okay, thank you.

**2. App – Finding / Installing**

Now, I’d like to talk about mobile apps to help people make and listen to recordings of their appointments. Some of these questions might sound repetitive, but we want to be thorough.

Please think back to the time you were finding and installing the app.

- - How did that go?
  - What if anything made it hard for you to find and install the app?
    - Did anything else make it difficult?
  - What if anything made it easier to find and install the app?
    - Was anything else helpful?
  - Can you think of anything that could help [mentioned difficulties]?
  - Is there anything else you’d like to tell us about your experience finding and installing the app?

**3. App - Recording**

Now, I’d like to ask about making a recording of the visit with [appointment provider] with the app.

- - How did that go?
  - What if anything made it hard to make the recording?
    - *If video visit*: Do you think having a video visit made it harder to make a recording compared to an in-person visit?
      - How so?
    - Did anything else make it hard?
  - What if anything made it easier to make the recording?
    - Was anything else helpful?
  - Can you think of anything that could help [mentioned difficulties]?
  - Is there anything else you’d like to tell us about your experience making a recording?

**4. App – Sharing, Listening *skip if didn’t make* *a recording***

Now, I’d like to ask about how you’ve used the recording.

Have you tried to access the recording in any way? Tell me more.

- - What if anything made it harder or easier to access the recording?
    - Anything else?

Who else if anyone did you share the recording with?

- - What if anything made it harder or easier to share the recording?
    - Anything else?

Have you listened to the recording? (In other words, played it back, reviewed it)

*If yes:*

- - How was your experience listening to the recording?
  - What if anything made it harder or easier to listen to the recording?
    - Anything else?
  - Can you think of anything that could help [mentioned difficulties]?
  - Is there anything else you’d like to tell us about your experience listening to the recording?

*If no:*

- - What was the main reason you didn’t listen to the recording?
    - Do you have any other reasons?

**5. Recording apps *skip if didn’t make* *a recording***

Do you agree with the goal to help men with prostate cancer make recordings of their appointments with an app? Please tell me more.

*Still ask the following questions even if nobody listened to the recording, since there are still potential benefits like 1) patients might pay better attention to the doctor if a recording is being made, or 2) they feel reassured that there is a recording they can listen to in the future.*

- - Was there anything about making or listening to the recording that helped you?
    - *If yes:* How did it help you?
    - *If no:* What are the reasons it didn’t help you?
    - Was there anything about the recording that helped others?
  - Did the recording help you make decisions about treating the prostate cancer?
    - *If yes:* How did it help you? Did that end up making a difference in your treatment decision?
    - *If no:* What are the reasons it didn’t help you?
  - What if anything made it hard for you to use the recording in a helpful way?
    - Did anything else make it difficult?
  - What if anything made it easy for you to use the recording in a helpful way?
    - Was anything else helpful?
  - Can you think of anything that could help [mentioned difficulties]?

**5. Other**

I have a few remaining questions now.

- - Did you have any concerns, problems, or discomfort about the app or the recording process?
    - *If yes:* Tell me more.
  - Are there any changes you would suggest to the app or the instructions we gave you (other than what you already mentioned?)

*If the participant has told you something interesting about audio recording but did not elaborate and you have time, please follow-up back to it here*

- - I want to go back to something you said earlier …

Here’s my final question. Let’s pretend that you had a magic wand that you could wave and make everything exactly the way you want. What would you put in place at UCSF so that you and everyone who wanted to could easily make audio recordings of their oncology visits?

**5. Disposition**

Thank you so much for taking the time to share your thoughts with me today. Before we end, is there anything else you would like to comment on that we haven’t yet discussed today?

Do you have any questions?

Thank you.

# **Document 3. Provider interview guide**

**1. Experience**

First, I’d like you to think back to your last appointment in which a patient recorded an appointment. *If cannot remember, skip to section 2.*

[Anchoring] To help you anchor this visit, can you tell me when it was and whether this was a Zoom or in-person visit?

How was that recording experience for you?

Did the recording get in the way of the appointment? How so?

Did you feel uncomfortable? How so?

Anything else you’d like to comment about that experience?

**2. Benefits**

Next, let’s talk about your thoughts on audio recordings overall.

What is your general opinion of patients having audio recordings of their oncology appointments?

What do you think are the benefits of audio recordings?

What evidence are you aware of surrounding the benefits of audio recordings for cancer patients?

In what ways do you think audio recordings meet the needs for your patients?

What benefits do you see patients making their own recordings using an app? (as opposed to doctor making it for them, or using a digital recorder and not an app).

Has there ever been a case in which a recording was particularly beneficial for a patient or you?

If yes: please tell me more [want details].

**3. Risks and Barriers**

Next, let’s talk about potential risks and barriers of audio recordings.

What do you think are the risks of patients having audio recordings of their oncology appointments? (Risks for patients, the doctor, health care system)

Has there ever been a case in which a recording had negative effects on the patient, you, or others?

If yes: please tell me more [want details].

What barriers do patients face when trying to record visits?

What do you think we can do to help patients overcome these barriers?

**4. Other**

Now, I’d like your feedback on our specific intervention in mencore-2. What we did was before an upcoming visit, we sent patients instructions on recording their visit, set up a training call before the appointment, and sent a text message reminder before the visit. In the future, we’d like to expand audio recordings across the UCSF cancer center.

What kinds of changes to this intervention do you think are needed for audio recordings to be delivered routinely in the GU and other cancer center clinics?

Now, think at the UCSF cancer center level and higher up, what kinds of changes to our intervention do you think are needed for success? (e.g., changes in **UCSF/state/national/public** **culture**, **policies, laws**, etc.)

What barriers do you see for UCSF to implement this sort of intervention across the cancer center?

How would you feel about the intervention being implemented routinely at UCSF?

Do you have any other comments?

**6. Disposition**

Thank you so much for taking the time to share your thoughts with me today.

Do you have any questions?

Thank you

# **Table 3. COnsolidated criteria for REporting Qualitative research**

| **Domain 1: Research team and reflexivity** |  |
| --- | --- |
| **Personal Characteristics** |  |
| 1. Interviewer/facilitator  Which author/s conducted the interview? | Patient/caregiver interviews: ND and EH  Provider interviews: SS |
| 2. Credentials  What were the researcher's credentials? | All interviewers had BA degrees. Researchers DK and JB involved in qualitative analysis have an MD and PhD, respectively |
| 3. Occupation  What was their occupation at the time of the study? | ND, EH, and SS: research assistant  DK: oncologist-scientist  JB: scientist |
| 4. Gender  Was the researcher male or female? | ND, EH, and SS: Female  DK and JB: Male |
| 5. Experience and training  What experience or training did the researcher have? | ND and EH (trained by DK in conducting semi-structured interviews), SS (trained by DK in conducting semi-structured interviews; 3 years prior experience in qualitative analysis), DK (coursework in qualitative research and implementation science; 5 years experience conducting qualitative research), JB (20 years experience conducting qualitative research and implementation science) |
| **Relationship with participants** |  |
| 6. Relationship established  Was a relationship established prior to study commencement? | Patient/caregiver interviews: Some patient participants were patients of DK. The rest of the patients and caregivers had no relationship with the research team, including ND, EH, and SS.  Provider interviews: All providers were colleagues of DK. The rest of the providers had no relationship with the research team, including SS. |
| 7. Participant knowledge of the interviewer  What did the participants know about the researcher? | The research team informed participants about the purpose of the study, that it was for research funded by the Conquer Cancer Foundation, and ethical approval had been granted. Participants reviewed and provided informed consent prior to procedures. Participants knew about the researchers’ roles in the research.  Provider participants had greater knowledge about DK’s research in consultation audio recordings, and several had consented to being recorded as part of the initial mencore pilot study and were authors in that study. |
| 8. Interviewer characteristics  What characteristics were reported about the interviewer/facilitator? | ND, EH, and SS were applying to medical school to become physicians at the time of the study. These may be sources of bias. |
| **Domain 2: study design** |  |
| **Theoretical framework** |  |
| 9. Methodological orientation and Theory  What methodological orientation was stated to underpin the study? | Ottawa Decision Support Framework and the Critical Incident Technique for interview guides  Critical Incident Technique and Rogers’ Diffusion of Innovations theory for qualitative analysis |
| **Participant selection** |  |
| 10. Sampling  How were participants selected? | Patient/caregiver interviews: consecutively. The research team first reviewed upcoming clinical schedules for potentially eligible participants. The team asked their oncologist whether they plan on discussing docetaxel at the upcoming appointment, whether they allow the research team to approach the participant, and whether they consent to be recorded.  Provider interviews: all UCSF genitourinary medical oncology providers (physicians and nurse practitioners). |
| 11. Method of approach  How were participants approached? | Patient/caregiver interviews: consecutively. The research team approached participants either in-person or by e-mail with telephone follow-up, 3-14 days prior the appointment.  Provider interviews: verbally and by e-mail. |
| 12. Sample size  How many participants were in the study? | Patient/caregiver interviews: 30 patients and 10 caregivers.  Provider interviews: 9 providers. |
| 13. Non-participation  How many people refused to participate or dropped out? Reasons? | Patients: none refused to participate.  Providers: 7 providers refused to participate for unknown reasons. |
| **Setting** |  |
| 14. Setting of data collection  Where was the data collected? | Interview data were collected remotely by telephone or video conference (Zoom). Participants were at home at the time of the interview. Researchers were at their workplace. |
| 15. Presence of non-participants  Was anyone else present besides the participants and researchers? | Caregivers had the option of joining the patient interviews. |
| 16. Description of sample  What are the important characteristics of the sample? | See Table 1. |
| **Data collection** |  |
| 17. Interview guide  Were questions, prompts, guides provided by the authors? Was it pilot tested? | See attached interview guides that were created using the Ottawa Decision Support Framework and Critical Incident Technique. They were not pilot tested, but adjustments were made based on the first several participant interviews. |
| 18. Repeat interviews  Were repeat interviews carried out? If yes, how many? | No |
| 19. Audio/visual recording  Did the research use audio or visual recording to collect the data? | Audio recording using Zoom. |
| 20. Field notes  Were field notes made during and/or after the interview or focus group? | Field notes were made during and after interviews. |
| 21. Duration  What was the duration of the interviews or focus group? | 30-45 minutes, but some lasted up to 60 minutes. |
| 22. Data saturation  Was data saturation discussed? | Patient/caregiver interviews: Data saturation was defined as no new critical incidents or themes after two consecutive interviews. Data saturation was reached after 30 interviews.  Provider interviews: We did not assess for data saturation. |
| 23. Transcripts returned  Were transcripts returned to participants for comment and/or correction? | No. |
| **Domain 3: analysis and findings** |  |
| **Data analysis** |  |
| 24. Number of data coders  How many data coders coded the data? | SS, ND, EH and DK coded the data. |
| 25. Description of the coding tree  Did authors provide a description of the coding tree? | Open and axial coding were performed by either SS, ND, or EH using a codebook based on the interview guide, then secondary coding was performed by DK. Discrepancies were negotiated to consensus during biweekly meetings. |
| 26. Derivation of themes  Were themes identified in advance or derived from the data? | Themes were identified in advance based on the codebook, and additional emergent themes were added based on ongoing interviews. |
| 27. Software  What software, if applicable, was used to manage the data? | Microsoft Excel, Microsoft Word, and Atlas.ti |
| 28. Participant checking  Did participants provide feedback on the findings? | No |
| **Reporting** |  |
| 29. Quotations presented  Were participant quotations presented to illustrate the themes / findings? Was each quotation identified? e*.g. participant number* | Yes |
| 30. Data and findings consistent  Was there consistency between the data presented and the findings? | Yes |
| 31. Clarity of major themes  Were major themes clearly presented in the findings? | Yes, in the results. |
| 32. Clarity of minor themes  Is there a description of diverse cases or discussion of minor themes? | No, in the results. |

#

# **Figure 2. Participant Flow Diagram**


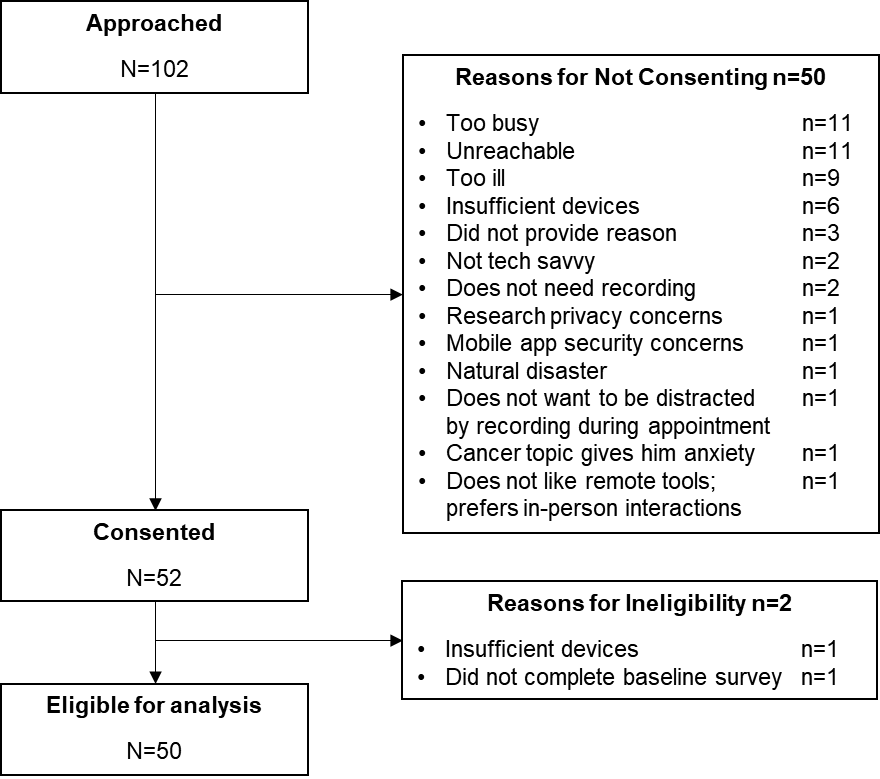


This figure illustrates application of eligibility and reasons for withdrawal or being unevaluable.

# **Table 4. Comparison of Evaluable versus Non-Responding or Unevaluable Participants’ Baseline Characteristics**

| **Characteristic** | **Evaluable (n=50)** | **Non-Responding or Unevaluable (n=52)** |
| --- | --- | --- |
| Mean age (years) ^a^ | 75 | 73 |
| Race/ethnicity ^b^ |  |  |
| White, Non-Hispanic | 39 (78%) | 35 (67%) |
| Asian, Non-Hispanic | 6 (12%) | 5 (10%) |
| Black, Non-Hispanic | 4 (8%) | 7 (13%) |
| White, Hispanic | 1 (2%) | 0 |
| Unknown / Declined | 0 | 5 (10%) |

^a^ Not statistically different based on independent two-sample t test.

^b^ Not statistically different based chi-squared test of proportion who are White, Non-Hispanic versus not.

# **Table 3. Patient- ,Caregiver-, and Provider-reported Benefits to Self-Administered Recordings**

| **Category** | **Specific Patient Benefits** |
| --- | --- |
| Communication | Help communicate with family by sharing recording after attending appointment alone |
|  | Appreciate provider's tone of voice |
| Decisions | Help with treatment decisions |
| Feelings | Feel empowered and in control |
|  | Provide reassurance that there is an accurate, unbiased record |
|  | Increase confidence in decision by reminding facts discussed by provider |
|  | Process own emotions, so feels more prepared for future appointments |
| Information | Clarify key information communicated by provider, like chemotherapy duration and outcomes |
|  | Revise information from provider that was missed, misheard, forgotten, or misremembered at time of appointment |
|  | Catches information not written down in notes taken by patient |
|  | Resolve information discrepancies heard by patient versus caregiver |
|  | Process information, like bad news |
|  | Look up medical jargon and clinical trial terminology |
|  | Prompt follow-up questions |
| Actions | Prompt need to schedule appointments or procedures that were forgotten |
| **Category** | **Specific Provider Benefits** |
| Feelings | Feel reassured that recording helps patient retain critical information |
|  | Receive gratitude from patient for allowing to be recorded |
| Information | Use more thoughtful words during appointment |
